# Insights into the prediction of the liquid density of refrigerant systems by artificial intelligent approaches

**DOI:** 10.1038/s41598-024-53007-1

**Published:** 2024-01-29

**Authors:** Huaguang Li, Alireza Baghban

**Affiliations:** 1 Intelligent Manufacturing College, Qingdao Huanghai University, Qingdao, 266427 Shandong China; 2 Process Engineering Department, National Iranian South Oilfields Company (NISOC), Ahvaz, Iran

**Keywords:** Energy science and technology, Engineering, Mathematics and computing

## Abstract

This study presents a novel model for accurately estimating the densities of 48 refrigerant systems, categorized into five groups: Hydrofluoroethers (HFEs), Hydrochlorofluorocarbons (HCFCs), Perfluoroalkylalkanes (PFAAs), Hydrofluorocarbons (HFCs), and Perfluoroalkanes (PFAs). Input variables, including pressure, temperature, molecular weight, and structural groups, were systematically considered. The study explores the efficacy of both the multilayer perceptron artificial neural network (MLP-ANN) and adaptive neuro-fuzzy inference system (ANFIS) methodologies in constructing a precise model. Utilizing a comprehensive dataset of 3825 liquid density measurements and outlier analysis, the models achieved R^2^ and MSE values of 0.975 & 0.5575 and 0.967 & 0.7337 for MLP-ANN and ANFIS, respectively, highlighting their remarkable predictive performance. In conclusion, the ANFIS model is proposed as an effective tool for estimating refrigerant system densities, particularly advantageous in scenarios where experimental measurements are resource-intensive or sophisticated analysis is required.

## Introduction

To create an economical refrigeration cycle for low temperatures, it is imperative to possess a thorough understanding of the thermodynamic characteristics of refrigerant systems, such as liquid density^1–4^. Despite the extensive amount of experimental data available in the literature, there are still incongruities among various datasets^5,6^. Therefore, it is crucial to calculate the thermodynamic characteristics of these substances for application in any scenario where experimental data is unavailable^7,8^.

Before the 1980s, in the refrigeration industry, the main refrigerants were hydrochlorofluorocarbons (HCFCs) and chlorofluorocarbons (CFCs)^9–11^. Since on January 1, 1996 production or use of CFCs was barred by Montreal Protocol (MP) so the quantity of CFCs decreased briskly^12,13^. Because of obstruction, which the MP placed on CFCs, HCFCs were replaced with CFCs in different industries^14^. Although HCFCs have decreased ozone layer damage, they still pose contamination issues and are planned to be removed by 2030. Hydro fluorocarbons (HFCs) because of very low ozone layer damage, coincidence to HCFCs and CFCs in terms of physical properties, limited existence in the atmosphere, minor flammability or nonflammable and economical were used as a substitution of CFCs and HCFCs^15^. Due to the serious global warming effect of HFCs, HFEs were introduced as a new generation of refrigerants by RITE^16,17^. Also, there are some compounds that have the potential of using refrigerant fluids such as perfluoroalkylalkanes (PFAAs) and perfluoroalkanes (PFAs)^18^. We do not have detailed studies that predict the thermodynamic properties of refrigerants by theoretical methods; so every author uses a special equation and method to forecast the thermodynamic properties of the refrigerant systems^19,20^. As the authors are cognizant, almost all of these attempts have been confined to the finite systems, and we don't have a systematical work for testing the qualification of each method^21–23^. The liquid density of refrigerants calculated applying 14 correlations and 4 equations of state (EOS) by Nasrifar and Moshfeghian^24^. Lugo et al. worked on aqueous solutions (secondary refrigerants) and suggested another method for calculating many of their thermophysical properties^25^. By forecasting the density of the HAs, HFEs, and their chemical mixtures, Scalabrin and his colleagues suggested density with a three-parameter model dependent on corresponding states^26^. Maftoon-Azad et al. inspected analytical EOS for predicting the density of HCFC and HFC refrigerants (compressed liquids)^27^ and for predicting the volumetric behavior of just six refrigerants they used the Ihm-Song-Mason equation^28^. In 2005, Goharshadi and Moosavi founded the density 3 HFEs and 11 HFCs and HCFCs^29,30^. Also, they applied this EOS for finite refrigerants. Generally, the previous equations or correlations of the state some adjustable parameters in addition to critical constants. For modeling the various thermodynamic properties, ANNs (Artificial Neural Networks) can be a convenient substitution^31,32^. A neural network is contrived from a large number of interconnected neurons that the organization of this neurons connection determines the structure of a network^33–35^. To achieve an overall desired behavior of the network, the arrangement of the connection strengths is controlled by the learning algorithms^36,37^. In other words, if you learn the relationship between output and input vectors, the ANN is a useful algorithm to prospect each function that has a limited number of discontinuities^38^. Hence, for modeling of the nonlinear treatment of chemical properties, the ANN is an appropriate technique^39,40^.

Recently, effective attempts have been made depending on the GC (group contribution) approach attached to the ANN model to develop the prediction models. Many researchers applied the ANN-GCM method for calculating the thermodynamic properties of various substances^41,42^. Namely, some properties of ionic liquids have been estimated with ANN-GCM methods such as melting point, density, thermal decomposition temperature, glass transition temperature, heat capacity, surface tension and viscosity^43–53^. Furthermore, this approach can be applied to anticipate the temperature at which flammability limits occur for organic compounds, the solid vapor pressure of pure compounds, the enthalpy of sublimation for organic compounds (at 298 K), the flash point temperature, and the vaporization enthalpy of organic compounds. It is also useful for predicting the densities of hydrocarbon systems, the specific volume of polymeric systems, and forecasting the density of liquid alkali metals^47,54–61^.

Since the last decade, for accurate prediction of the refrigerant systems thermodynamical properties, some bounded efforts have been made to extend ANN models. Chouai et al. utilized the ANN method for PVT depiction of R32, R134a, and R143a in the temperature range of 240 to 340 K and pressure up to 20 Mega Pa^62^. Also, Mohebbi et al. used GA-ANN (a neural network relied on the genetic algorithm) for 6 mixed and 14 pure refrigerants to estimate the saturated liquid density^5^. Their model can predict this parameter with a mean absolute error (%) of 3.53 and 1.46 for mixed and pure refrigerants, respectively. The latest research in this area was conducted by Moosavi and his colleagues, who used ANN-GCM model to predict density of refrigerants^18^. Their model has the ability to predict this parameter with average absolute deviation of 0.28 at testing phase.

The purpose of this study is to estimate the liquid densities for various refrigerant mixtures at a wide range of pressures and temperatures with three intelligent approaches that contain MLP-ANN, and ANFIS. To develop these models, a large data set is used. Then, different statistical methods are used to evaluate and analyze the obtained models. Additionally, an outlier analysis will be performed to identify suspected points.

## Theory

### MLP-ANN

ANN is a method that is acquired to rely on biological NNs created by a collection of interconnected nodes that admitted as artificial neurons. This method has the proficiency in handling signals sent by links between the nodes. Principally, every artificial neuron uses a non-linear aggregate of inputs of the neuron for determining the outputs. As well as, a weight parameter could be used to decrease or increase the strength of signals at the connections. Commonly, for a node, we have three categories of the activation functions that used to obtain the output from a determined collection of inputs (Eqs. 1–3):

Linear function:

1 $$h\left(y\right)=ySPS : id\colon\colon\upepsilon 1$$

Sigmoid function:

2 $$g\left(y\right)=\frac{1}{1+{e}^{-y}}$$

Hyperbolic tangent function:

3 $$k\left(y\right)=\frac{{e}^{y}-{e}^{-y}}{{e}^{y}+{e}^{-y}}SPS : id\colon\colon\upepsilon 3$$

Furthermore, an effective factor in the definition of ANNs is the bias term. MLP-ANN includes hidden, input and output layers and it is a feed-forward type of ANN. The activation function of this kind of ANN’s nodes is non-linear, and a back propagation training (BPT) approach is applied in this model^63,64^.

### ANFIS

Primarily, Zadeh introduced the notion of Fuzzy Logic (FL), which had the capability to arrange outputs on a spectrum from completely false to completely true. In contrast, classical logic is only able to arrange conclusions as either true or false^65^. Consociate of linguistic rules of if–then and principals of fuzzy logic developed the model. Using the basics of fuzzy logic helps us to touch a transparent development process and outputs with more accuracy. Coupling ANN and fuzzy logic make it possible to get precise solutions for extraordinarily non-linear systems^66,67^. ANFIS is created of integrating from fuzzy logic and ANN. Mamdani and Takagi–Sugeno are two structures for FIS^68–70^. Logical explanation in progress of fuzzy if–then rules used in the first FIS type, but the next type of FIS generates the if–then rules rely on the performance of accessible empirical data. The Takagi–Sugeno type inference system used in the ANFIS method to demonstrate the non-linear reliance of variables^70^. An if–then rule is applied in a generic ANFIS structure for ${Y}_{1}$ and ${Y}_{2}$ (input parameters) as follow (Eqs. 4–7):

4 $$if\, {Y}_{1} \,is\, {C}_{1} \,and\, {Y}_{2} \,is\, {D}_{1} \,then\, { g}_{1}={p}_{1}{Y}_{1}+{q}_{1}{Y}_{2}+{s}_{1}$$

5 $$if\, {Y}_{1} \,is\, {C}_{2} \,and\, {Y}_{2} \,is\, {D}_{2} \,then\, {g}_{2}={p}_{2}{Y}_{1}+{q}_{2}{Y}_{2}+{s}_{2}$$

6 $$if\, {Y}_{1} \,is\, {C}_{1} \,and\, {Y}_{2} \,is\, {D}_{2} \,then\, {g}_{3}={p}_{3}{Y}_{1}+{q}_{3}{Y}_{2}+{s}_{3}$$

7 $$if\, {Y}_{1} \,is\, {C}_{2} \,and\, {Y}_{2} \,is\, {D}_{1} \,then\, {g}_{4}={p}_{4}{Y}_{1}+{q}_{4}{Y}_{2}+{s}_{4}$$

In these equations, $g$ shows the output parameters and respectively ${C}_{i} and{ D}_{i}$(*i* = 1, 2) are fuzzy sets for ${Y}_{1} and {Y}_{2}$. Generally, this structure has 5 layers. The initial layer for fuzzification utilizes the membership function to convert input data into linguistic terms. In this investigation, the GM is employed and is defined as follows:

8 $${O}_{i}^{1}=\upgamma \left(P\right)={\text{exp}}\left[-\frac{1}{2}\frac{\left(P-Q\right)}{{\Lambda }^{2}}\right]$$

In Eq. (8), Q represents the center of the Gaussian distribution,${\Lambda }^{2}$ refers to the variance and *O* is the output of the layer. For getting the most accurate model, GM should be optimized. In the second layer, by computing the commonly referred to firing strength parameters, it becomes possible to assess the dependability of the preceding components (Eq. 9):

9 $${O}_{i}^{2}={v}_{i}={\upgamma }_{Ai}\left(P\right){\upgamma }_{Bi}\left(Q\right)$$

Moreover, in the third layer, the normalization of estimated firing strengths has been carried out (Eq. 10):

10 $${O}_{i}^{3}=\overline{{v }_{i}}=\frac{{v}_{i}}{{\sum }_{i}{v}_{i}}$$

In Eq. (11) for output parameter the linguistic terms are defined (fourth layer):

11 $${O}_{i}^{4}=\overline{{v }_{i}}{g}_{i}=\overline{{{\text{v}} }_{i}}\left({p}_{i}{Y}_{1}+{q}_{i}{Y}_{2}+{s}_{i}\right)$$

where $q$, ${r}_{s}$ and ${p}_{i}$ are linear parameters for optimization. Eventually, in the fifth layer all of the rules associated to an output will be appear in the following formula (Eq. 12):

12 $${O}_{i}^{5}={\sum }_{i}\overline{{v }_{i}}{g}_{i}=\frac{{\sum }_{i}{v}_{i}{g}_{i}}{{\sum }_{i}{v}_{i}}$$

## Model development

### Preprocessing procedure

This paper shows three strategies such as ANFIS and MLP-ANN are applied to estimate density rely on molecular mass (Mw), pressure (P), the structural groups and temperature (T). The structural groups used as input parameters are shown in Table 1. The computational tools and platforms employed for model development and evaluation were crucial components of our methodology. Specifically, MATLAB (version 2020) served as the primary computational environment for implementing and assessing the ANFIS and MLP-ANN strategies applied to estimate density. The choice of MATLAB was driven by its versatility, extensive toolboxes for neural network implementation, and widespread use in scientific research.

**Table 1:** Categories of structural groups examined in MLP-ANN and ANFIS models.

− CF_3_	CF_2_Cl_2_	− CF_2_H
− CCl_2_H	CF_3_Cl	− CH_2_F
− CF_2_Cl	CF_4_	− CFH −
− CFClH	CCl_2_FH	− CF_2_ −
− CCl_2_F	CF_2_ClH	− CH_2_ −
− CH_3_	CF_3_H	− O −
CCl_3_F	CF_2_H_2_	

Furthermore, the dataset employed for modeling purposes, consisting of 3825 data points, is meticulously sourced and extensively referenced throughout the manuscript^16,71–86^. These data points, carefully selected from reputable and relevant literature, form the foundation of our study and contribute to the robustness and validity of our modeling approach*.* Testing and training subsets got from collected data. 25% of data are applied for testing, and 75% of data are occupied for training the recommended models. Normalization of data was performed by Eq. (13)^87^:

13 $${\Delta }_{Norm}=2\frac{D-{D}_{min}}{{D}_{max}-{D}_{min}}-1$$

where the $\Delta$ is the parameter value. Norm, max, and min stand for the normalized, maximum, and minimum values, respectively. The normalized data spans from − 1 to 1. Density is the output of the model, and the input variables are the other four parameters that were mentioned earlier.

### Model development

#### MLP-ANN

Following parameter shows the general output parameter for this model (Eq. 14):

14 $${Y}_{\Sigma }=\sum_{i=1}^{n}\left({W}_{3i}\frac{1}{1+{e}^{-\left({x}_{i}{W}_{i}\right)}}\right)+{b}_{3}$$

In Eq. (14), ${W}_{i,}, {W}_{i,3}$ and $n$ are respectively, the weight vector for neurons and for output layer neurons, and the number of hidden layer neurons. Also, ${b}_{3}$ is the bias term.

Additionally, decreasing the differences between real and estimated data gives us the optimum output parameter (using the ANN structure). The minimization occurs with regulating weight and bias parameters. In this task, we utilize the error function determined in Eq. (15):

15 $${S}_{\Sigma }=\sum_{j}\sum_{i}\left({r}_{i}^{j}-{o}_{i}^{j,l}\right)$$

**Figure 1:**
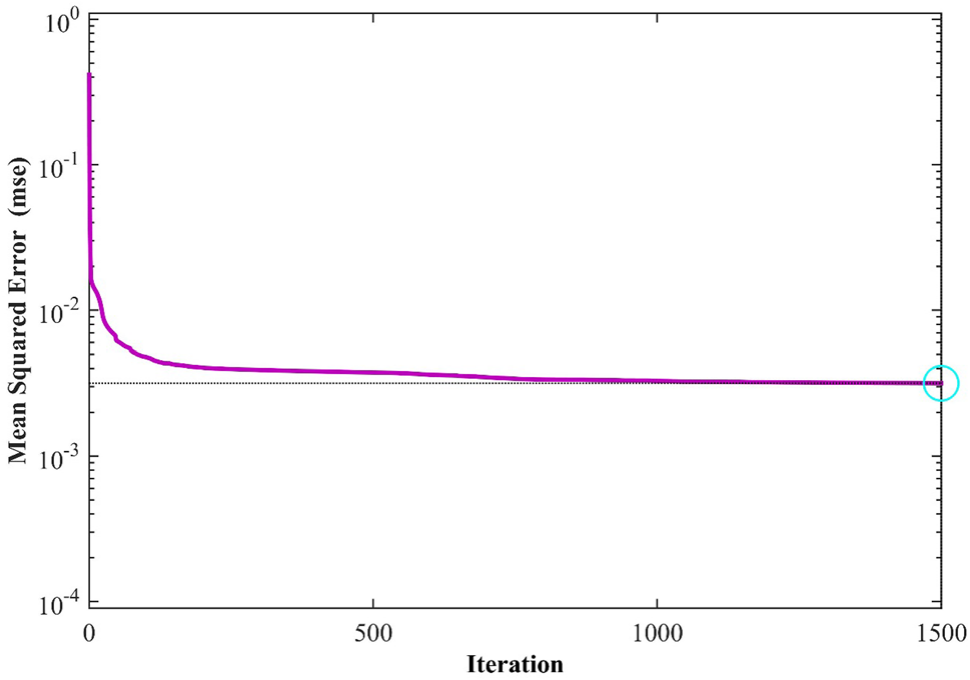
MLP-ANN's performance based on Mean Squared Error across various iterations of the LM algorithm.

The notation ${r}_{i}^{j}$ represents the ith actual output for the jth data point, while ${o}_{i}^{j,l}$ denotes the output of the ith neuron in the first layer, where j is the data point index in the training dataset. Applying the Levenberg–Marquardt algorithm causes optimization. Moreover, Fig. 1 shows the performance of the utilized network relies on the MSE calculated data by using MLP-ANN.

#### ANFIS

**Figure 2:**
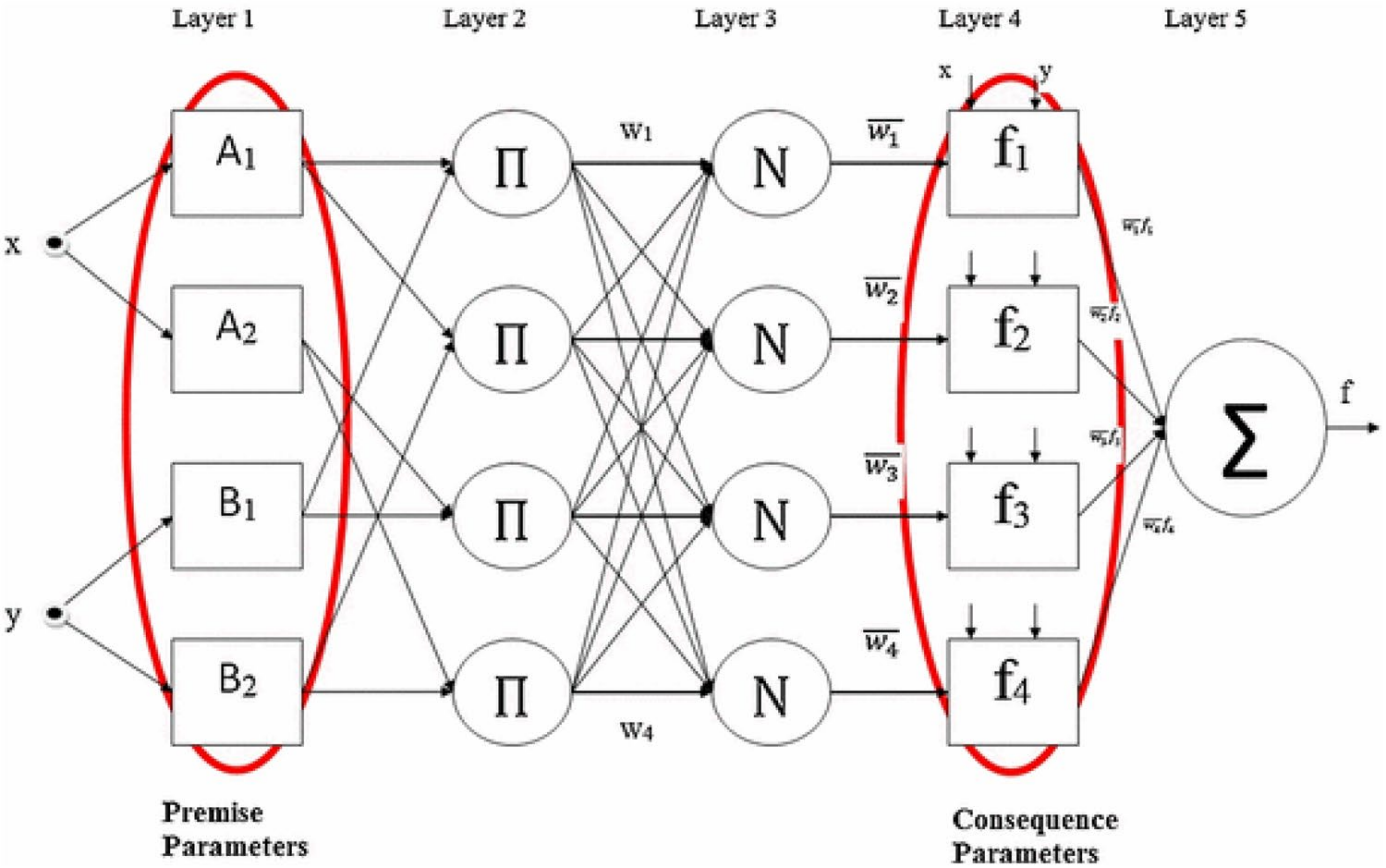
The ANFIS structure.

Figure 2 shows the diagram of a generic ANFIS includes two variables as input. Training the proposed ANFIS takes place by utilizing a genetic algorithm (GA). Equation (16) determines the whole parameters of this model that depends on the number of variables (${N}_{v}$), the number of clusters (${N}_{c}$) and the number of MF parameters (${N}_{MF}$):

16 $${P}_{Tot}={N}_{c}.{N}_{v}.{N}_{MF}$$

**Figure 3:**
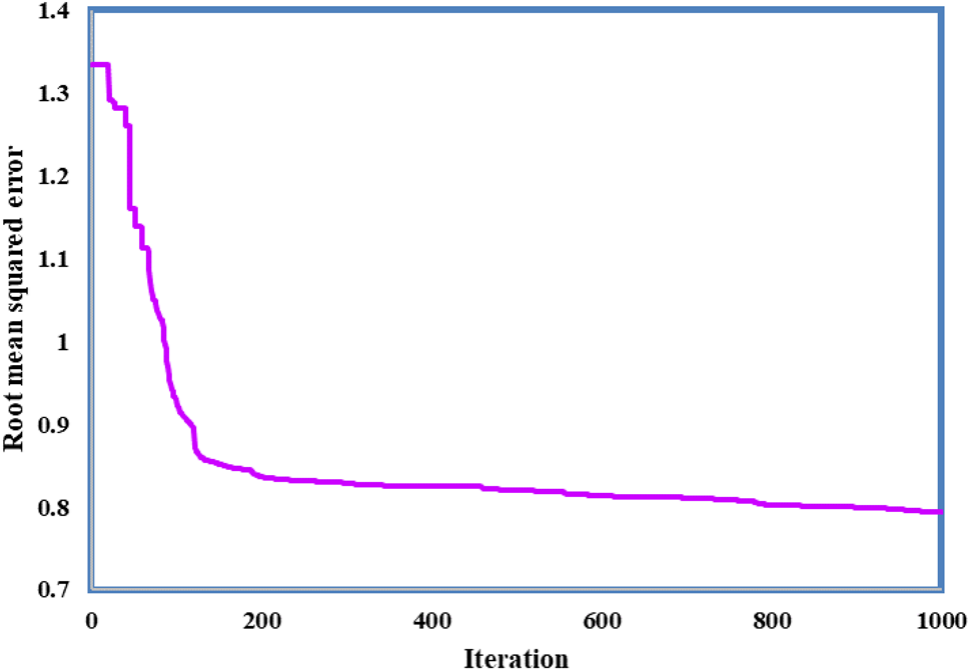
Performance of ANFIS during the training stage employing the Particle Swarm Optimization approach.

In this manuscript, the MF utilized is the GM function. $Z and {\sigma }^{2}$ are the MF parameters. Pressure (P), temperature (T), molecular mass (Mw), and the structural groups are input variables. So, for 480 ANFIS parameters, the total number was obtained. For the GA algorithm used in achieving the optimum parameters of this model, the cost function is the RMSE between the real and estimated data. Figure 3 denotes the RMSE values of each iteration.

### Models’ evaluation

For attaining to the precision of the predictive model's, root mean squared error (RMSE), coefficient of determination (R^2^), mean squared error (MSE), standard deviation (STD) and AARD are acceptable statistical criteria’s. Following equations are the mathematical definition of mentioned criteria (Eqs. 17–21)^88^:

17 $${\text{EMeanSqErr}}=\frac{1}{N}\sum_{i=1}^{N}{({D}_{i}^{exp.}-{D}_{i}^{cal.})}^{2}$$

18 $${AARD}_{{\text{Perc}}}=\frac{100}{N}\sum_{i=1}^{N}\frac{\left|{D}_{i}^{exp.}-{D}_{i}^{cal.}\right|}{{D}_{i}^{exp.}}$$

19 $${\text{STDev}}={\left(\frac{1}{N-1}\sum_{i=1}^{N}{\left({D}_{i}^{exp.}-{D}_{i}^{cal.}\right)}^{2}\right)}^{0.5}$$

20 $${\text{RootMeanSqErr}}={\left(\frac{1}{N}\sum_{i=1}^{N}{\left({D}_{i}^{exp.}-{D}_{i}^{cal.}\right)}^{2}\right)}^{0.5}$$

21 $${R}_{Coef}^{2}=1-\frac{{\sum }_{i=1}^{N}{\left({D}_{i}^{exp.}-{D}_{i}^{cal.}\right)}^{2}}{{\sum }_{i=1}^{N}{\left({D}_{i}^{exp.}-\overline{{D }^{exp.}}\right)}^{2}}$$

where $\overline{{D }^{exp.}}$denotes the mean experimental output value (density), $exp.$ and ${\text{cal}}.$ are an abbreviation of the experimental and calculated values, and N denotes the quantity of data points.

## Results and discussion

The presented strategies were hired to determine the density of various refrigerant systems by considering pressure (P), temperature (T), the structural groups, and molecular mass (Mw) as input parameters. More information about these intelligent models is brought in Table 2. Using the MLP-ANN model, density values are better estimated than other model (ANFIS). This fact has been proven by the statistical analysis given in the evaluation section of the models.

**Table 2:** Additional information about models trained for density estimation.

ANFIS	
Category	Value/remark
MF	Gaussian
Number of MF parameters	480
Number of clusters	10
Amount of data utilized for training	2926
Amount of data utilized for testing	975
Optimization technique	PSO
Population size	85
Iteration	1000
C_1_	1
C_2_	2
MLP-ANN	
Number of input neuron layers	23
Number of hidden neuron layers	20
Number of output neuron layers	1
Activation function for hidden layer	Logsig
Activation function for output layer	Purelin
Data used for training	2926
Data used for testing	975
Maximum iterations	1500

**Figure 4:**
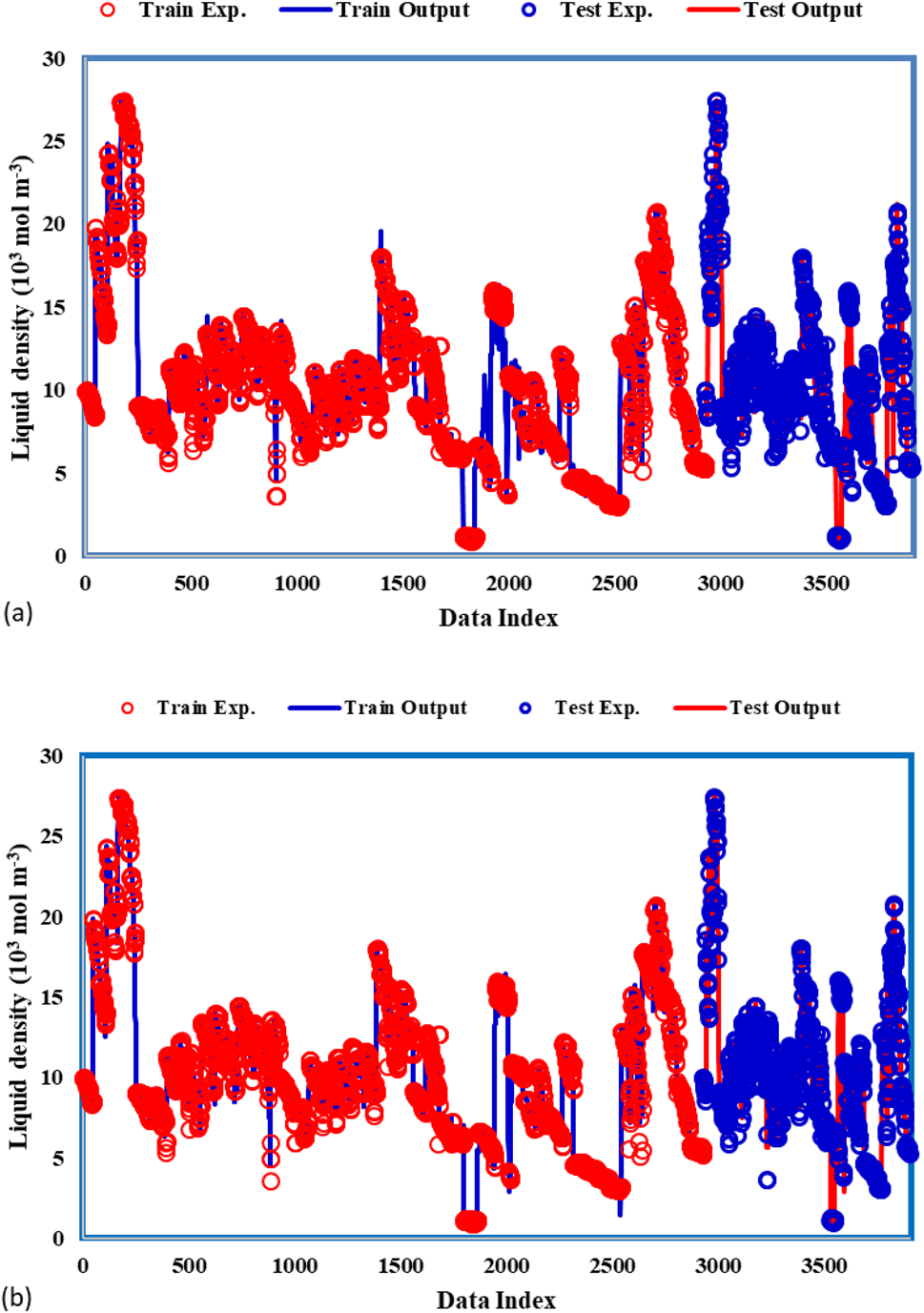
Comparison between estimated density values and experimental data using different models: (**a**) ANFIS, and (**b**) MLP-ANN.

To assess the efficacy of the models employed in this study, we employed various graphical methods. Figure 4 illustrates the plots comparing experimental and predicted density values for each model. Notably, the MLP-ANN model emerges as the most precise performer in density prediction, demonstrating a superior alignment between predicted and actual values. This graphical representation provides a clear visual insight into the predictive accuracy of the models, with the MLP-ANN model showcasing particularly commendable performance.

**Figure 5:**
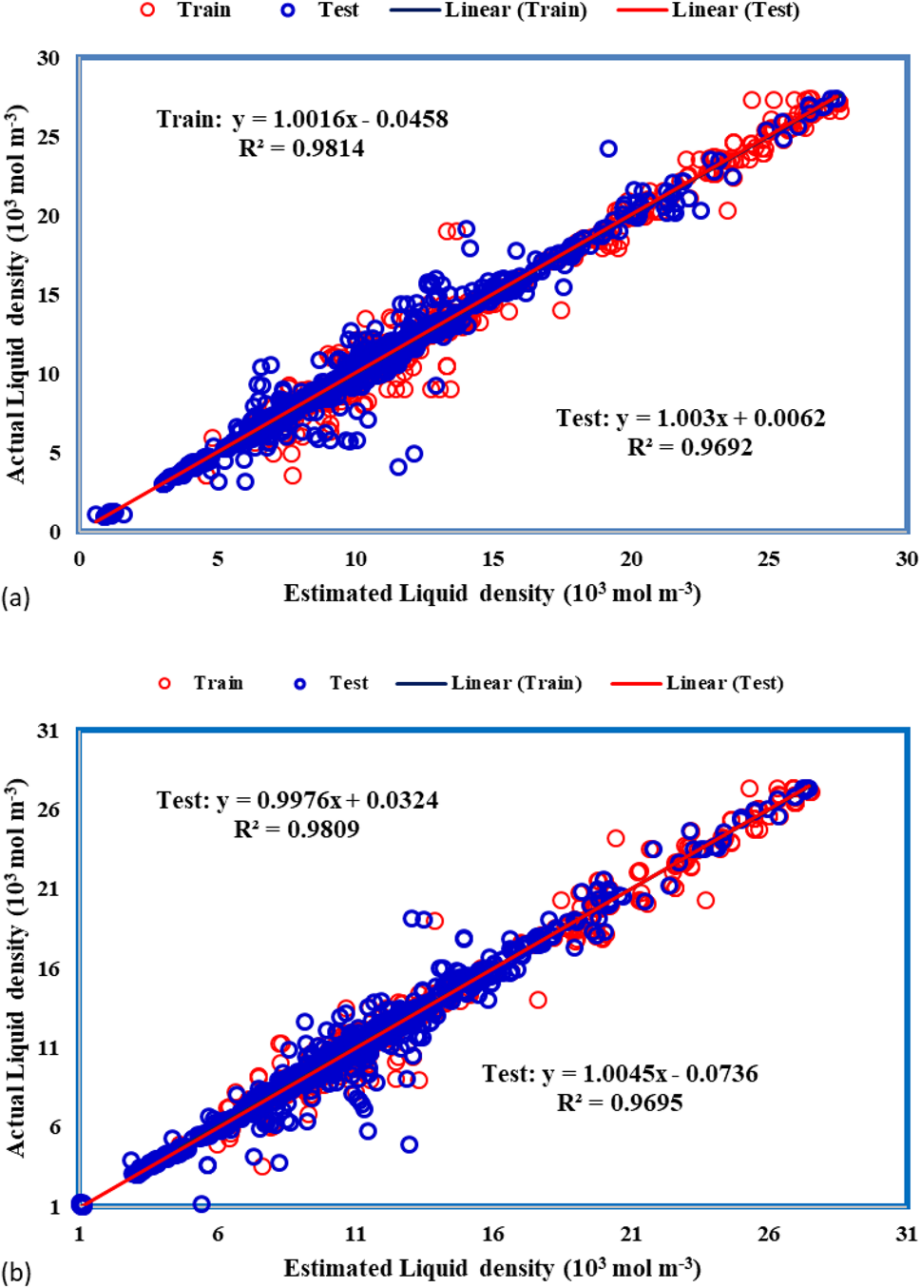
Regression diagram to estimate density using different models in the training and testing steps; (**a**) ANFIS, (**b**) MLP-ANN.

Regression plots between experimental versus predicted density values are shown in Fig. 5. The best fitting lines are obtained by using linear regression between the real and estimated values (Fig. 5a,b).

**Figure 6:**
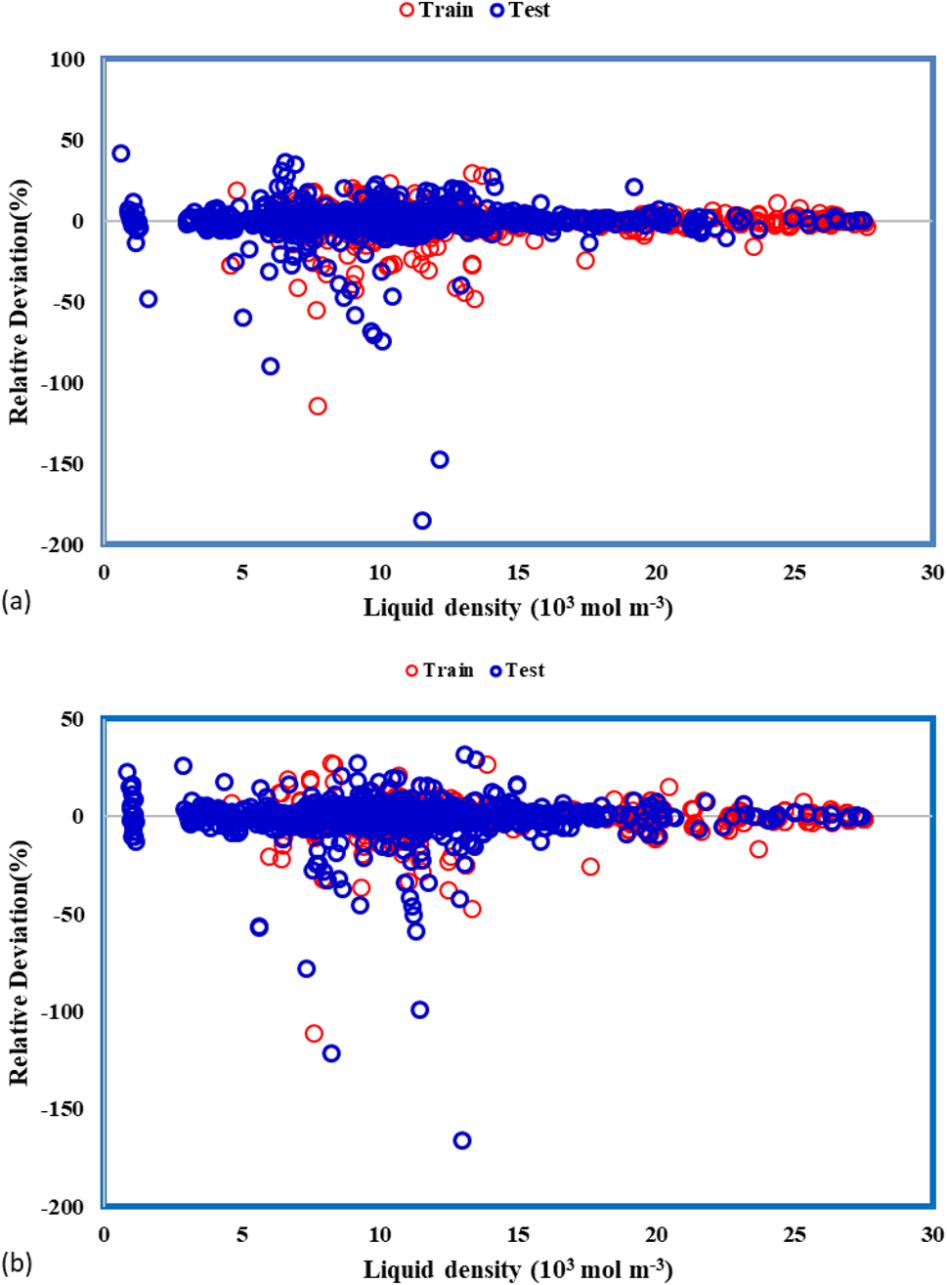
Percentage relative deviation of testing and training data with various models: (**a**) ANFIS, and (**b**) MLP-ANN.

The relative deviations of the estimated and real data are shown in Fig. 6. It is apparent that the least deviation is related to the MLP-ANN model.

As a matter of fact, this is because of the accumulation of data points around the zero line. MRE values for MLP-ANN and ANFIS are 4.751 and 5.068, respectively. Additionally, for understanding the capability of these models in the prediction of the density values the statistical error analyses are done. These analyses are brought in Table 3.

**Table 3:** Assessing the effectiveness of the proposed models through statistical analysis.

Model	Dataset	R^2^	MRE (%)	MSE	RMSE	STD
ANFIS	Train	0.966	5.233	0.7598	0.8717	0.7574
	Test	0.969	4.575	0.6555	0.8096	0.7006
	Total	0.967	5.068	0.7337	0.8096	0.7436
MLP-ANN	Train	0.976	4.671	0.5260	0.7252	0.5987
	Test	0.969	4.990	0.6522	0.8076	0.6881
	Total	0.975	4.751	0.5575	0.8076	0.6222

### Outlier detection

The precision of the models put forward is significantly impacted by the actual data employed in the segment dedicated to model development^89^. In order to ensure the robustness of our models, locating and removing a set of data points exhibiting distinct behavior from the rest of the dataset, referred to as outliers, is regarded as a crucial step in enhancing the reliability of models^90^. The leverage analysis is used in addition to standardized residuals implementation to determine potential outliers. By plotting standardized residuals (R) versus hat values (H), William’s plot, outliers are detected. This multifaceted approach allows us to thoroughly assess the data points that might disproportionately influence the model outcomes. Equation (22) is used to calculate diagonal Components of the hat matrix, which are expressed as hat values and are used in the identification of feasible/suitable regions.

22 $$H=X{\left({X}^{T}X\right)}^{-1}{X}^{T}$$

Taking into account n as the number of data points and k as the number of input parameters, ${\text{X}}$ represents a $\left(n\times k\right)$ matrix. This matrix is instrumental in evaluating the influence of each data point on the model. Warning leverage and cut-off values on the horizontal and vertical axis make a squared area called the feasible region. Below equation gives the warning leverage:

23 $${H}^{*}=\frac{3\left(k+1\right)}{n}$$

**Figure 7:**
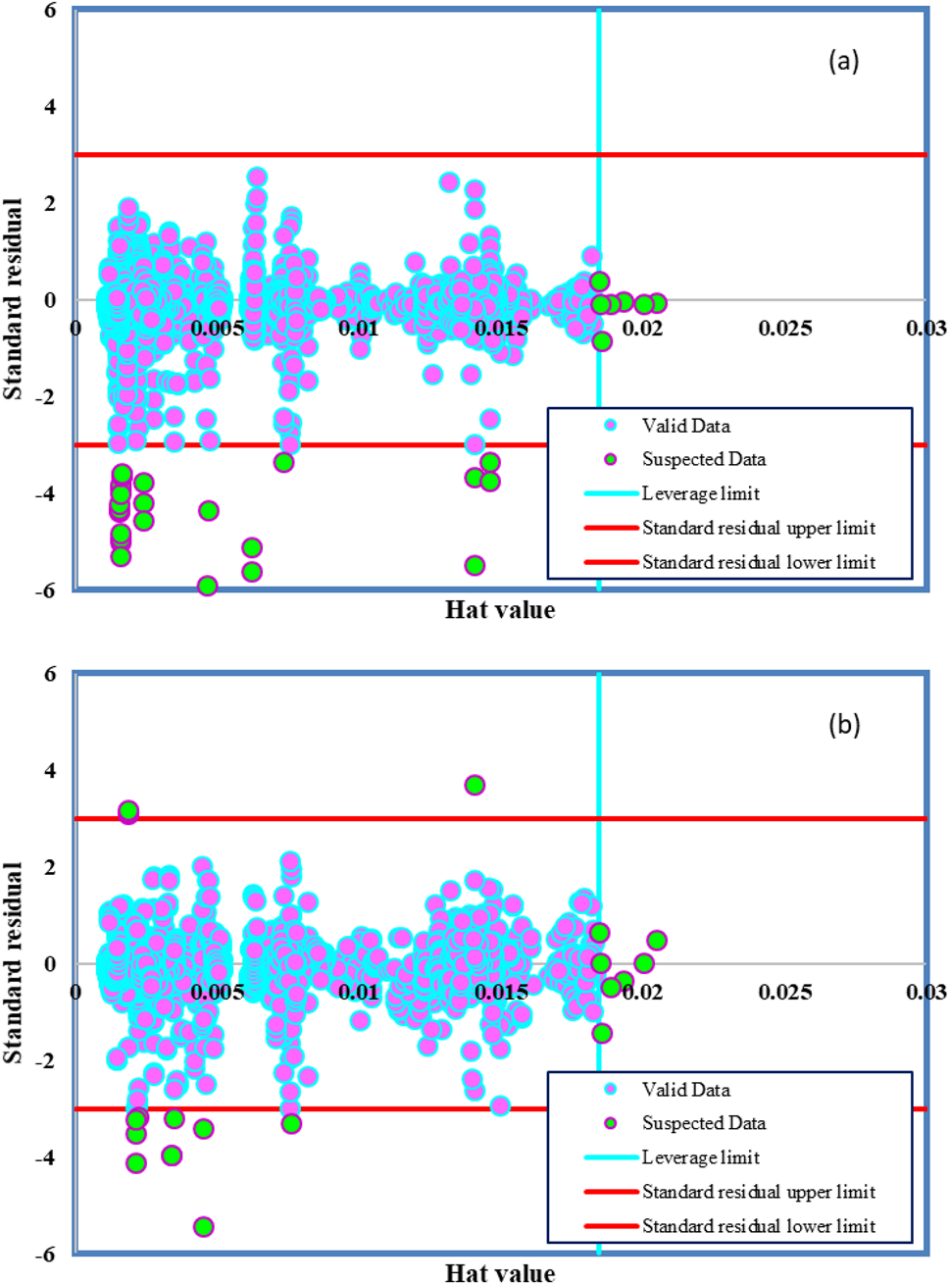
Identification of potentially questionable datasets for various models: (**a**) ANFIS, (**b**) MLP-ANN.

This calculated warning leverage is pivotal in setting thresholds to identify potential outliers. Typically, the threshold value for R is deemed to be 3. Values beyond the boundaries of the feasible region are treated as outlier data. By meticulously considering these calculated parameters, our approach offers a comprehensive method for identifying and addressing outliers in the dataset. Figure 7 shows William’s plot. According to this Figure, 27 and 17 points of the ANFIS and MLP-ANN approaches are placed outside of the feasible region.

While the presented models, MLP-ANN and ANFIS, exhibit remarkable predictive performance in estimating the densities of various refrigerant systems, it is essential to acknowledge certain limitations in the current study. One limitation is the reliance on a specific dataset comprising 3825 data points. Although the dataset is meticulously sourced from reputable literature, its scope may not cover all possible scenarios and variations in refrigerant properties. Additionally, the models are developed based on the selected input parameters, including molecular mass, pressure, structural groups, and temperature. The exclusion of certain relevant parameters or the consideration of additional factors could potentially impact the models' generalizability to a broader range of refrigerant systems. Furthermore, the outlier analysis conducted in this study identified specific data points that deviate from the predicted trends. While these outliers were carefully addressed, their presence underscores the sensitivity of the models to anomalous data. Future research endeavors could explore ways to enhance the robustness of the models by incorporating more diverse datasets, exploring additional input parameters, and implementing advanced outlier detection techniques. Moreover, continuous refinement of the models through ongoing validation against experimental data will contribute to their reliability in practical applications.

## Conclusions

In summary, the prediction of densities for 48 refrigerant systems was facilitated through the utilization of two intelligent models, incorporating crucial parameters such as molecular mass (Mw), pressure (P), structural groups, and temperature (T). The superior performance of the MLP-ANN approach over the ANFIS model, demonstrated by consistently lower error values, has been highlighted by the findings. Transcending its theoretical significance, the research harbors practical implications of particular relevance to scientists and engineers engaged in the design of economically viable low-temperature refrigeration cycles. The accuracy demonstrated by the MLP-ANN model establishes it as a valuable tool, one that effectively guides the optimization of system performance and steers the development of energy-efficient and cost-effective refrigeration technologies. These implications, indicative of the broader impact, underscore the study's contribution to advancements in the field of refrigeration system design. The findings not only deepen the understanding of the intricacies involved but also actively contribute to the evolution of methodologies, offering insights that shape the trajectory of progress in refrigeration technology.

## Data Availability

The data that support the findings of this study are available from the corresponding author, Alireza Baghban, upon reasonable request.

## Abbreviations

HFEs: Hydrofluoroethers
RMSE: Root mean squared error
HCFCs: Hydrochlorofluorocarbons
PFAAs: Perfluoroalkylalkanes
HFCs: Hydrofluorocarbons
PFAs: Perfluoroalkanes
MLP: Multilayer perceptron
BPT: Back propagation training
ANN: Artificial neural network
GA: Genetic algorithm
CFCs: Chlorofluorocarbons
RBF: Radial basis function
RITE: Research institute of innovative technology for the earth
FL: Fuzzy logic
MP: Montreal Protocol
AARD: Average absolute relative deviation
HAs: Halogenated alkanes
STD: Standard deviation
GC: Group contribution
R^2^: Coefficient of determination
EOS: Equations of state
MF: Membership function
LM: Levenberg–Marquardt
MSE: Mean Squared Error
RDP: Relative deviation percentage
ANFIS: Adaptive neuro-fuzzy inference system
